# Posttraumatic Proximal Radioulnar Synostosis after Closed Reduction for a Radial Neck and Olecranon Fracture

**DOI:** 10.1155/2018/5131639

**Published:** 2018-02-19

**Authors:** Patrick R. Keller, Heather A. Cole, Christopher M. Stutz, Jonathan G. Schoenecker

**Affiliations:** ^1^Department of Orthopaedics and Rehabilitation, Vanderbilt University Medical Center, 4202 Doctors' Office Tower, 2200 Children's Way, Nashville, TN 37232-9565, USA; ^2^Texas Scottish-Rite Children's Hospital for Children, 2222 Welborn Ave., Dallas, TX 75219, USA; ^3^Department of Pathology, Vanderbilt University Medical Center, 4202 Doctors' Office Tower, 2200 Children's Way, Nashville, TN 37232-9565, USA; ^4^Department of Pharmacology, Vanderbilt University Medical Center, 4202 Doctors' Office Tower, 2200 Children's Way, Nashville, TN 37232-9565, USA; ^5^Department of Pediatrics, Vanderbilt University Medical Center, 4202 Doctors' Office Tower, 2200 Children's Way, Nashville, TN 37232-9565, USA; ^6^Vanderbilt Center for Bone Biology, Vanderbilt University Medical Center, 4202 Doctors' Office Tower, 2200 Children's Way, Nashville, TN 37232-9565, USA

## Abstract

Posttraumatic proximal radioulnar synostosis (PPRUS) is a severe complication of radial head and neck fractures known to occur after severe injury or operative fixation. Cases of PPRUS occurring after minimally displaced, nonoperatively treated radial neck injuries are, by contrast, extremely rare. Here, we present a pediatric case of PPRUS that developed after a nonoperatively treated minimally displaced radial neck fracture with concomitant olecranon fracture. While more cases are needed to establish the association between this pattern of injury and PPRUS, we recommend that when encountering patients with a minimally displaced radial neck fracture and a concomitant elbow injury, the rare possibility of developing proximal radioulnar synostosis should be considered.

## 1. Introduction

Fractures of the proximal radius are among the most problematic of elbow injuries due to the high incidence of complications including nerve injury, osteonecrosis, stiffness, and decreased range of motion [1]. Posttraumatic synostosis of the proximal radioulnar joint (PRUJ) is recognized as a severe complication of radial head and neck fractures because it severely limits range of motion. It is most commonly associated with displaced radial neck injury and/or operative intervention [1–22], especially after percutaneous fixation or open reduction and internal fixation maneuvers are performed [1]. Thus, in keeping with current standard of practice [1], radial neck fractures with <30° angulation and less than 3-4 millimeters of translation are usually best treated with closed reduction and casting. For these cases, closed reduction and casting almost universally provides an optimal reduction and avoids major complications, including PPRUS [2, 3, 23–25]. However, though extremely rare, cases of PPRUS after minimally displaced nonoperatively treated radial neck injury have occurred. In our review of the literature, there was only one case report of PPRUS that developed after closed reduction and immobilization for a radial neck fracture with concomitant elbow dislocation. Here, we present a novel case of PPRUS following a minimally displaced, nonoperatively treated radial neck fracture with concomitant olecranon fracture in a pediatric patient.

## 2. Case

**Figure 1:**
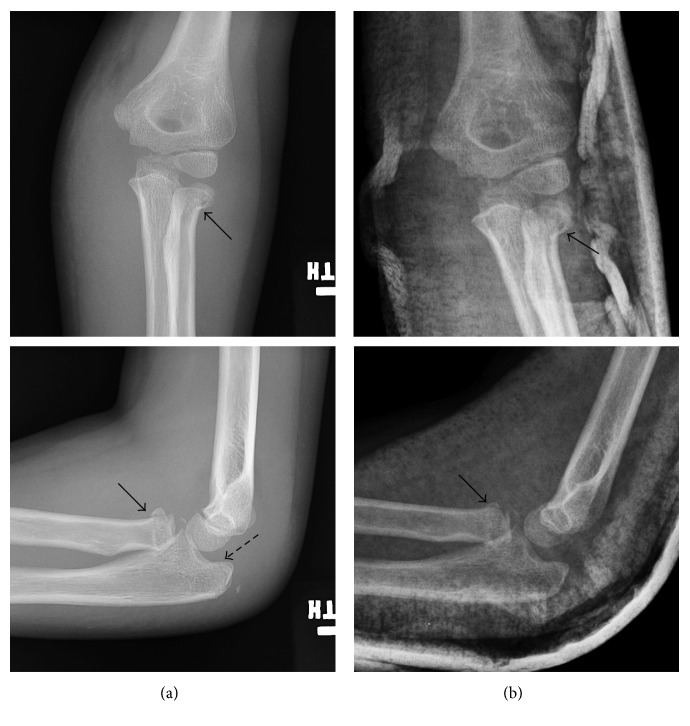
Left elbow before (a) and after (b) attempted closed reduction. Initial injury AP and lateral radiographs (a) demonstrate a radial neck fracture with 20–30° angulation (solid arrows in (a)) and a nondisplaced olecranon fracture (hatched arrow in (a)). Post-closed reduction AP and lateral radiographs (b) are partially obscured by plaster splint but demonstrate no change in alignment of radial neck (solid arrows in (b) compared to (a)).

**Figure 2:**
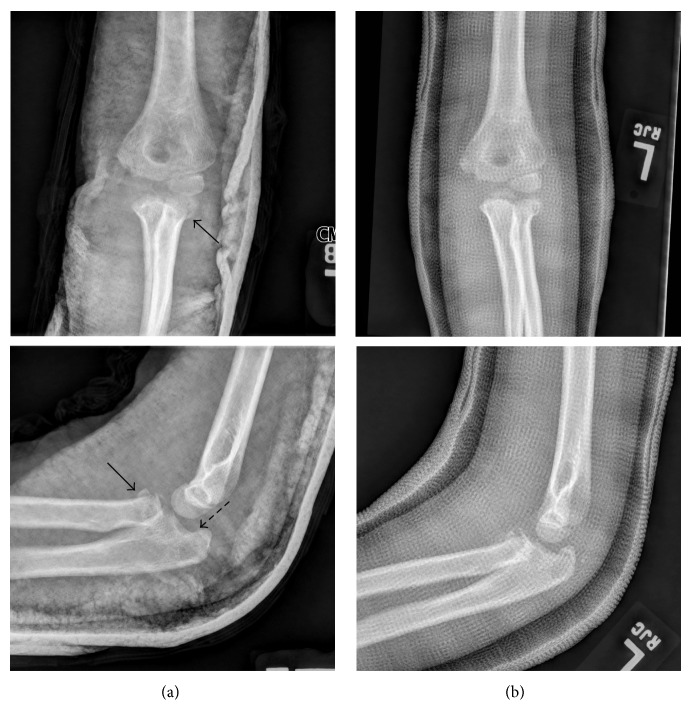
Left elbow radiographs one (a) and two (b) weeks after injury. One-week AP and lateral radiographs (a) are partially obscured by splint material but demonstrate radial neck fracture with less than 30° of angulation (solid arrows in (a)) and a nondisplaced olecranon fracture (hatched arrow in (a)). Splint was removed and cast placed at one week. Two-week AP and lateral radiographs (b) are partially obscured by overlying cast material but demonstrate no changes from prior radiographs.

The patient was an 8-year-old right-handed girl who fell from eight feet onto her outstretched and supinated left upper extremity. She immediately experienced elbow pain and swelling. In the emergency department, she was unable to move her elbow without significant pain. Initial radiographs showed a left radial neck fracture and a nondisplaced olecranon fracture (Figure 1(a)). Elbow range of motion was 40° shy of full extension to 110° of flexion, supination 15° shy of neutral and pronation of 30°. Reduction of the radial neck fracture was attempted via closed reduction under fluoroscopic guidance. Follow-up radiographs showed <30° angulation and 1 mm of translation of the radial head (Figure 1(b)). Her left arm was placed in a well-padded posterior A-frame splint. She was sent home in the splint and returned to clinic one week later. One week after injury, radiographs were taken and then her splint was removed. A long arm cast was placed (Figure 2(a)). Two weeks after injury, left elbow radiographs showed no change in alignment (Figure 2(b)).

**Figure 3:**
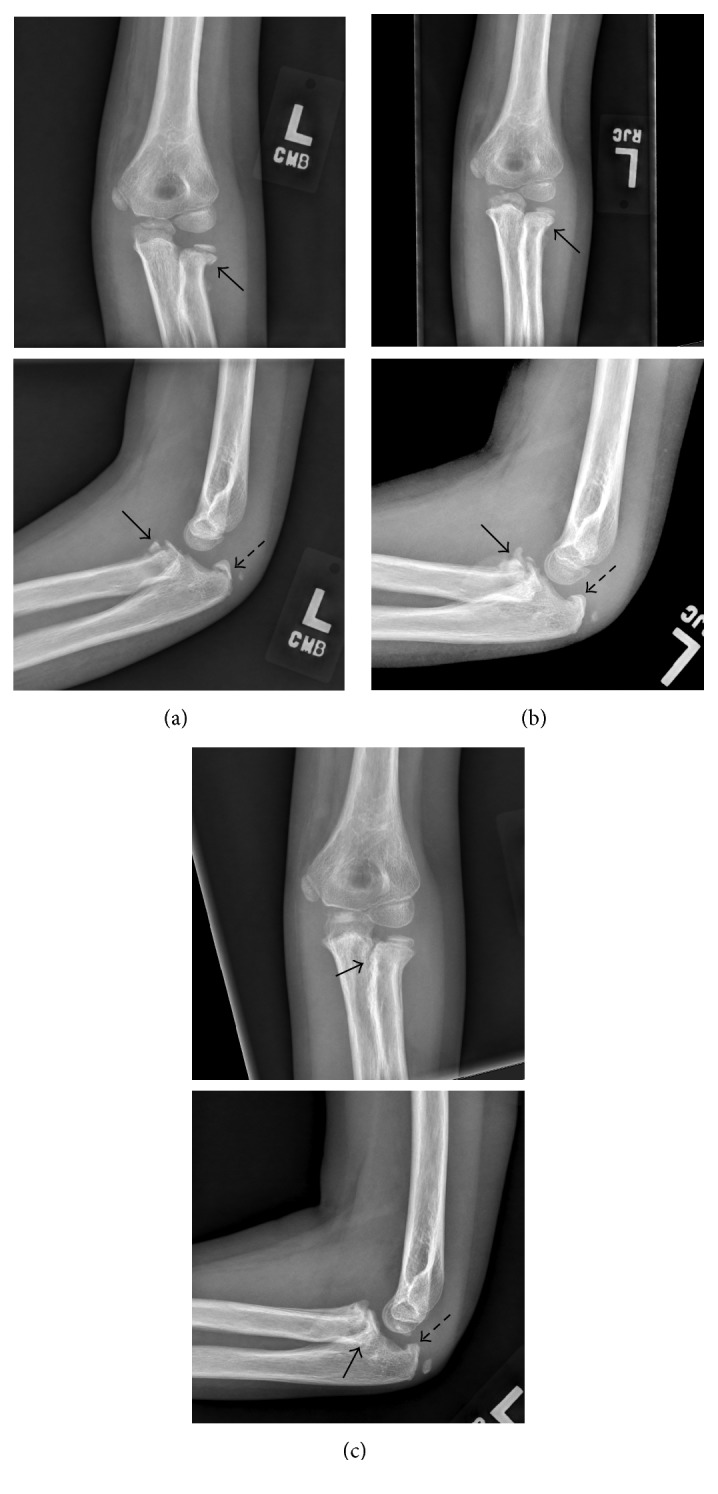
Left elbow radiographs three (a), four (b), and eleven (c) weeks after injury. Cast was removed at three weeks. Three-week AP and lateral radiographs (a) demonstrate a radial neck fracture with mild medial displacement and no change in alignment from prior (solid arrows in (a)) and a now-displaced olecranon fracture (hatched arrow in (a)). Four-week AP and lateral radiographs (b) demonstrate increased periosteal reaction about the radial neck fracture (solid arrows in (b)) with no change in alignment from prior and a healing olecranon fracture (hatched line in (b)). Eleven-week AP and lateral radiographs (c) demonstrate increased callus formation and synostosis about the medial aspect of the radial neck fracture (solid arrows in (c)) and a healed olecranon fracture (hatched arrow in (c)).

Three weeks after injury, the cast was removed and physical examination revealed full extension with 90° of flexion and a pronosupination arc of 40°, both limited by pain. Radiographs demonstrated minimal displacement of the olecranon fracture, but no change in radial neck fracture alignment (Figure 3(a)). She was prescribed range of motion exercises with the assistance of a physical therapist. Four weeks after injury, physical examination revealed pronosupination between −10° and 20°, with almost full pronation. She was without pain. Radiographs demonstrated healing radial neck and olecranon fractures (Figure 3(b)). Eleven weeks after injury, physical examination revealed extension to 90°, flexion to 30°, supination 15° from neutral, and pronation to 20° from neutral, all limited by pain. In addition to continued healing of fractures, radiographs demonstrated a new finding of synostosis at the PRUJ (Figure 3(c)). Continued physical therapy was recommended, and the family was informed that surgical resection of the synostosis may be needed to improve her range of motion. She was lost to follow-up for almost two years, at which point she returned to our clinic.

**Figure 4:**
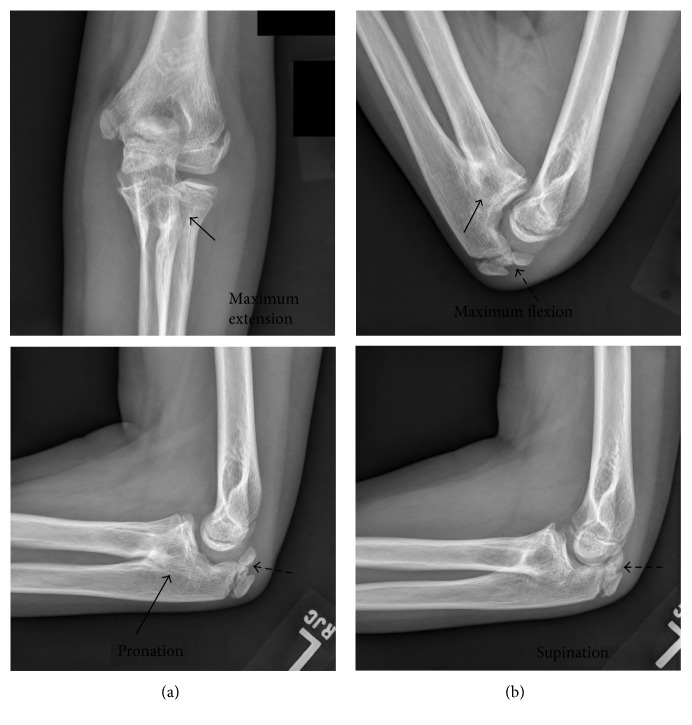
Left elbow radiographs at two years after injury. Maximum extension and pronation (a) and flexion and supination (b) are shown. On physical examination, she had full range of motion in extension and flexion, but pronosupination was entirely lost. All radiographs show synostosis of the proximal radioulnar joint (solid arrows in (a) and (b)) and fracture deformity from the previously healed proximal radial fracture. The olecranon fracture is healed (hatched arrows in (a) and (b)).

**Figure 5:**
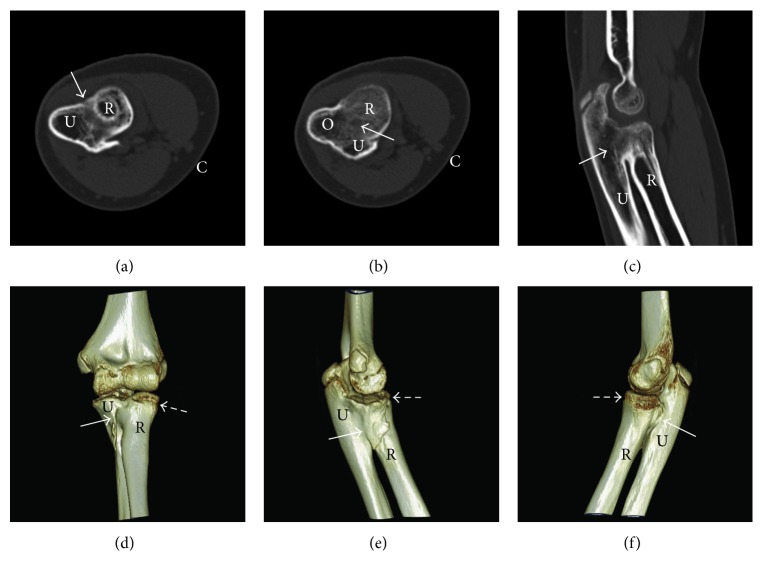
Left elbow CT scans (a–c) and CT 3D reconstruction (d–f) at two years six months after injury. Axial (a, b) and sagittal (c) CT views demonstrate synostosis of the proximal radioulnar joint, as do anterior (d), medial (e), and lateral (f) CT 3D reconstruction views (solid white arrows in all). The radial head fracture is healed (hatched white arrows in (d)–(f)). U = ulna; R = radius; O = olecranon; C = cubital fossa.

Two years after injury, physical examination of the elbow revealed 135° of flexion and full extension, but complete loss of pronosupination with the forearm fixed in 10° of supination. Radiographs demonstrated healed radial neck and olecranon fractures, and an extensive bony synostosis at the PRUJ (Figures 4(a) and 4(b)). At that time, surgical treatment of the synostosis was offered, but the patient was again lost to follow-up for six months. Two years six months after injury, she again returned to our clinic, and physical examination was unchanged from prior. CT scan (Figure 5) confirmed extensive bony synostosis at the proximal radioulnar joint. She was again offered a multitude of treatment options, including surgical resection, and family chose nonsurgical management with physical therapy. She has not returned to our clinic since.

## 3. Discussion

While past studies have described risk factors for and the incidence of PPRUS as it occurs after displaced injury or operative fixation for radial neck fractures [1–3, 10, 11, 14, 16–19, 22], this same information is lacking in regard to minimally displaced, nonoperatively treated injury. Our review of literature suggests that in cases of radial neck fracture treated by closed reduction, two factors increase risk of PPRUS: (i) increased radial head angulation or displacement [13] and (ii) concomitant elbow injuries [4].

Regarding the risk factor of increased severity of fracture, literature suggests that PPRUS tends to develop in displaced radial neck fractures (>45° angulation or >3-4 mm translation), especially in injuries that have been treated operatively [3, 11, 24, 26]. This feature of radial neck fractures is well known and has been recognized since the early 20th century. In 1933, Dr. John Bohrer described radial head and neck fractures in twenty adults and nine children, all of whom were treated conservatively. He found that PPRUS occurred only when there was “marked displacement” or “severe injury with displacement or comminution of the head of the radius.” By contrast, in cases of “slight displacement” or “slight trauma,” he observed “excellent results” [13]. Other studies, by and large, support the conclusion that minimally displaced nonoperatively treated radial neck fractures do not typically develop PPRUS [12, 27, 28]. This is in contrast to our patient who, although she sustained a minimally displaced nonoperatively treated injury, did have a concomitant elbow injury.

Much less is known about minimally displaced radial neck fractures that both (i) were treated conservatively and (ii) had associated elbow injuries (e.g., elbow dislocation or ulnar fracture). In one case series, Jones et al. [4] described a young girl who, after sustaining a radial neck fracture with associated elbow dislocation, was treated with closed reduction and immobilization for two weeks and then developed PPRUS. The displacement of her radial neck fracture was not described. Because of that, there is no way of discerning whether PPRUS was secondary to the associated elbow dislocation, the severity of the radial neck fracture, or a combination of both. However, our case report adds to the literature an example of PPRUS from a minimally displaced radial neck fracture that occurred in the setting of concomitant elbow injury (proximal ulnar fracture in our case), thus supporting the latter interpretation (combination) of increased risk in the Jones et al. case.

Our hypothesis is that in cases of minimally displaced nonoperatively treated proximal radius fracture with concomitant elbow injury, PPRUS forms because of disruption of periosteum from the transmission of energy from the wrist, to the radial neck, through the PRUJ, and finally to the ulna. In our patient's case, her pattern of injury can be classified as a Monteggia variant fracture; such fractures are characterized by an olecranon fracture with an associated radial neck fracture [2]. The most common mechanism of injury for Monteggia variant fractures is from fall on an outstretched supinated arm [3, 25]. Valgus torque is exerted on the elbow joint which, when combined with the force that is transmitted from the wrist up through the radial shaft, drives the radial head into the capitellum [2, 5, 20]. Because the radius and ulna articulate proximally in only one place, the PRUJ, injury to the radius will transmit the force to the ulna through the PRUJ. This increases the likelihood of injury to the periosteum at the PRUJ, predisposing the patient to development of PPRUS. This mechanism is supported by studies that have shown that injury (e.g., from trauma or surgery) stimulates pluripotential mesenchymal stem cells to differentiate into osteoblasts, which then lay osteoid which becomes heterotopic lamellar bone [18]. Further, injuries to the interosseous membrane of the forearm have the potential to serve as the nidus for heterotopic ossification and subsequent synostosis at any point along the membrane [19, 29].

This hypothesis does not exist in a vacuum, however, as several other etiologic mechanisms of PPRUS have been proposed in the past. These include arthrosis of the elbow joint space, ossification of elbow ligaments (including the annular and collateral ligaments), disruption of the joint capsule leading to capsular fibrosis [20], soft tissue contracture, ectopic calcification of surrounding soft tissue structures (including myositis ossificans of the brachialis muscle), failure of fracture healing [18], and bone fragments that inadvertently remain in the interosseous membrane [19]. It is possible that one or more of these other etiologic factors played a role in our patient's case. Thus, more case reports are needed to further develop and test our hypothesis.

## 4. Conclusion

Our case is a radiographically confirmed report of association between a minimally displaced, nonoperatively treated proximal radius injury and development of PPRUS over subsequent weeks. Critically, this occurred in a patient whose radial neck fracture (i) was minimally displaced, (ii) had a concomitant olecranon fracture, and (iii) was treated nonoperatively. This suggests that proximal radioulnar synostosis may be a rare part of the natural history of minimally displaced radial neck fractures in children, especially in cases of associated elbow injury. Therefore, we recommend that when encountering patients who sustain a radial neck fracture with associated elbow injuries, the clinician should consider the rare possibility of the patient developing proximal radioulnar synostosis and obtain closer follow-up with radiography and range of motion. Further work should strive to better establish the pattern of injury described herein, and whether certain types of concomitant elbow injuries in the setting of radial neck fracture are more associated with PPRUS than others.
